# Cultural Adaptation, Validation, and Primary Application of a Questionnaire to Assess Intentions to Eat Low-Glycemic Index Foods among Rural Chinese Women

**DOI:** 10.3390/ijerph17207577

**Published:** 2020-10-18

**Authors:** Mingshu Li, Fang Li, Qian Lin, Jingzheng Shi, Jing Luo, Qing Long, Qiping Yang, Yufeng Ouyang, Hanmei Liu, Rhonda C. Bell, Jia Guo

**Affiliations:** 1 Department of Nutrition Science and Food Hygiene, Xiangya School of Public Health, Central South University, Changsha 410078, China; limingshu@csu.edu.cn (M.L.); linqian@csu.edu.cn (Q.L.); luojing2546@csu.edu.cn (J.L.); yangqiping12@csu.edu.cn (Q.Y.); oyyf0102@csu.edu.cn (Y.O.); hanmeiliu@csu.edu.cn (H.L.); 2 Department of Epidemiology and Health Statistics, Xiangya School of Public Health, Central South University, Changsha 410078, China; lifang_csu@csu.edu.cn (F.L.); shijch@csu.edu.cn (J.S.); 3 Xiangya School of Nursing, Central South University, Changsha 410013, China; longqing226@163.com; 4 Department of Agricultural, Food and Nutritional Science, University of Alberta, Edmonton, AB T6G 2H6, Canada; Rhonda.bell@ualberta.ca

**Keywords:** Chinese version of the intentions to eat low-glycemic index foods questionnaire (CIELQ), cross-culture adaptation, validation, application, women with previous gestational diabetes mellitus (GDM), Hunan, rural area

## Abstract

Different lines of evidence indicate that knowledge of low-glycemic index (GI) foods and the practice of eating them play important roles in blood glucose management and preventing T2DM in women with prior gestational diabetes mellitus (GDM). According to the theory of planned behavior (TPB), intention is a critical factor in complying with health-related behaviors. However, an instrument for assessing the intention to eat low-GI foods is lacking in China. We aimed to (1) adapt and validate a Chinese version of the intentions to eat low-GI foods questionnaire (CIELQ) and (2) apply the CIELQ among rural Chinese women to explore the associations between CIELQ scores and glycemic status. A cross-sectional study was conducted on 417 nondiabetic, nonpregnant participants with a history of GDM in Hunan, China. After cultural adaptation and validation, the CIELQ was applied in a target population. Glycemic status, anthropometric variables, dietary intake, and physical activity were measured; a self-developed, standard questionnaire was applied to collect relevant information. The CIELQ showed good internal consistency; model fitness was acceptable based on the confirmatory factor analysis results. Awareness of the glycemic index was low among the study population. TPB factors were found to be associated with each other; education level and parents’ diabetes history were associated with specific factors. The score for instrumental attitude showed a positive association with the risk for a high level of the 2-h 75-g oral glucose tolerance test (odds ratio, OR = 1.330), while the score for perceived behavior control (PBC) showed a negative association with the risk for a high level (OR = 0.793). The CIELQ was determined to be a valid instrument for assessing the intention to eat a low-GI diet among the study population. The awareness of the GI was poor among the study population. The score for instrumental attitude showed a positive association with the risk of a high level on the 2-h 75-g oral glucose tolerance test (OGTT), and the score for PBC showed a negative association with the risk for a high level on OGTT.

## 1. Introduction

Gestational diabetes mellitus (GDM) is characterized by hyperglycemia diagnosed for the first time during pregnancy, and it is usually detected by routine testing during pregnancy, generally between 24 and 28 weeks [1]. GDM is one of the most common complications during pregnancy, with an estimated prevalence of 5.8–12.9% globally [2]. In China, GDM ranks first among maternal complications, with a prevalence is 14.8–23.0%, which has increased rapidly in recent decades [3,4]. GDM is associated with a higher incidence of fetal and maternal morbidity. In addition to the increased incidence of perinatal conditions, such as perinatal mortality, macrosomia, and preeclampsia [5], women with a history of GDM are at higher risks of subsequently developing type 2 diabetes mellitus (T2DM) [2,6]. Increasing lines of evidence suggest that nutrition therapy plays an important role in blood glucose management and preventing T2DM in women with prior GDM [7]. In particular, consuming a diet based on low-glycemic index (GI) foods results in better glycemic control than following conventional healthy dietary recommendations [8,9]. A meta-analysis conducted in 2019 that included 29 studies compared different dietary strategies for glycemic control among Chinese women with GDM [10]. Six of them compared a low-GI diet with standard dietary interventions and found that low-GI diets were associated with improved glycemic control. GI-based approaches have been proven to help improve the carbohydrate and overall diet quality, self-care practices, and metabolic outcomes among diabetes patients [11,12]. Thus, Chinese women with prior GDM may benefit from a diet emphasizing low-GI foods.

A few studies focused on pregnant women have identified a poor understanding of GDM [13]. A qualitative exploratory study assessed the awareness of GDM among rural Chinese women, and the authors reported a serious lack of knowledge, low level of risk awareness, and poor self-care behavior among the study group [14]. Few studies focused on the concept of the GI have been conducted on postpartum rural Chinese women with a history of GDM. Thus, for this population, there are no relevant documented data on awareness of the GI or on the use of a low-GI diet.

As mentioned above, dietary choice is crucial for preventing T2DM in women with prior GDM. Thus, it is important to analyze the factors related to the behavior of the target population. Health behavior theories such as the theory of planned behavior (TPB) provide a framework for understanding human behavior [15]. According to the TPB, three constructs influence intentions (INT): attitudes toward the behavior (ATT), subjective norms regarding the behavior (SN), and perceived behavior control (PBC); these intentions then influence actions [16]. The TPB is widely used to explain the associations between intentions to eat a between healthy diet and adherence behavior [17,18]. Eades and colleagues conducted a study using semi-structured interviews according to the TPB [19]. Their finding revealed that changing the diet was the first choice related to lifestyle changes during and after pregnancy among women with GDM; this practice was most commonly motivated by instrumental attitude, such as concern for the fetus and the desire to avoid taking medication to treat GDM. In addition, women who lived outside the main towns (i.e., with less PBC) reported less motivation for maintaining healthy practices (i.e., blood testing) postnatally. In summary, the TPB is suitable for explaining health-related behaviors among women with GDM.

Under the framework of the TPB, Watanabe and colleagues developed a tool to assess intentions to eat low-GI foods (IELQ) in adults with diabetes in Canada [20]. This questionnaire evaluated seven factors, including knowledge of the GI, instrumental attitude, experiential attitude, subjective norms, descriptive norms, PBC, and behavioral intention. They found that the IELQ had acceptable validity (factor loading range, 0.53–0.96) and reliability (Cronbach alpha range, 0.78–0.93), explaining 62.9% of the total variance in intentions to eat low-GI foods. These results indicate that IELQ is a valid tool for assessing TPB constructs that contribute to the intention to eat a low-GI diet in people with diabetes.

Nevertheless, this instrument was not culturally adapted for use in other countries; nor was a similar tool developed for assessing the intention to eat low-GI foods in rural Chinese women with a prior history of GDM. Because of the importance of intention in actually performing a health-related behavior according to the TPB, it is crucial to determine the intention of eating low-GI foods among a target population and explore the potential influence of this intention. Thus, we conducted this study to (1) adapt the IELQ and validate a Chinese version (CIELQ) to assess the intention to eat low-GI foods among Chinese women with GDM in rural areas and (2) explore the association between the CIELQ results and glycemic status.

## 2. Materials and Methods

### 2.1. Ethical Approval and Study Design

This study was conducted as part of the project “Effectiveness of a diabetes prevention programme for rural women with prior gestational diabetes mellitus,” which has been approved by the ethical committee of Xiangya Nursing School of Central South University (No. 2016034) and has been registered in the Chinese Clinical Trial Registry (ChiCTR1800015023). The present study data were drawn from the baseline survey used in the project mentioned above. According to the rule of thumb, 15–20 subjects are required for per predictor in a logistic regression model [21]. We assumed 10–15 predictors in this study, and considering a 20% non-response or drop-off rate, the range of the final sample size was determined to be 180 to 360.

### 2.2. Participant Recruitment and Enrollment

The baseline survey was carried out among a sample of 465 rural, nondiabetic, nonpregnant women with a history of GDM, from two geographical areas in rural Hunan, China, in 2017. The selection process for the setting and participants was described in our previous study [22].

**Figure 1:**
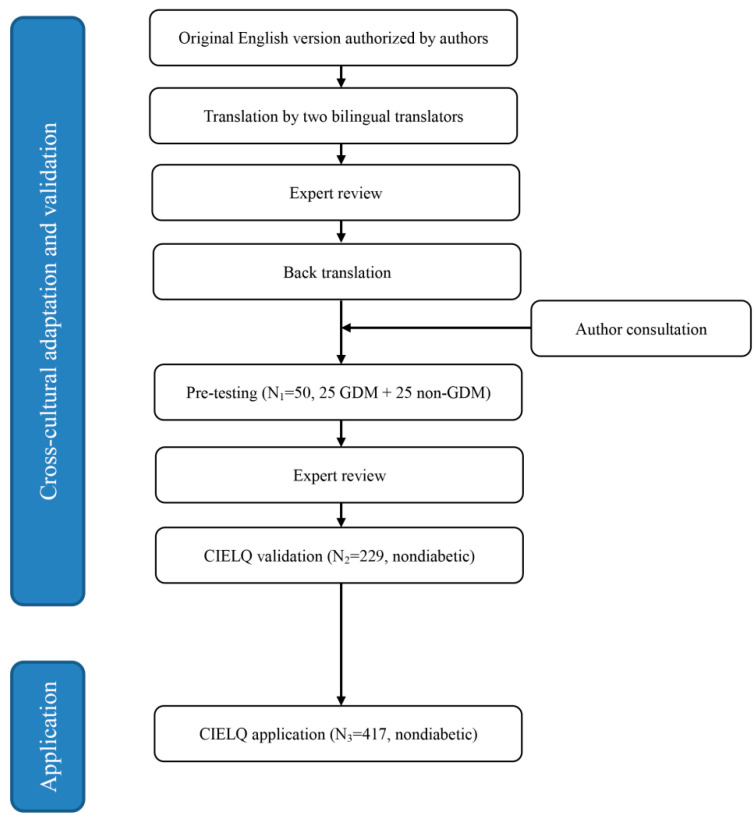
Flow chart of the cross-cultural adaptation and application among rural women with GDM history.

**Figure A1:**
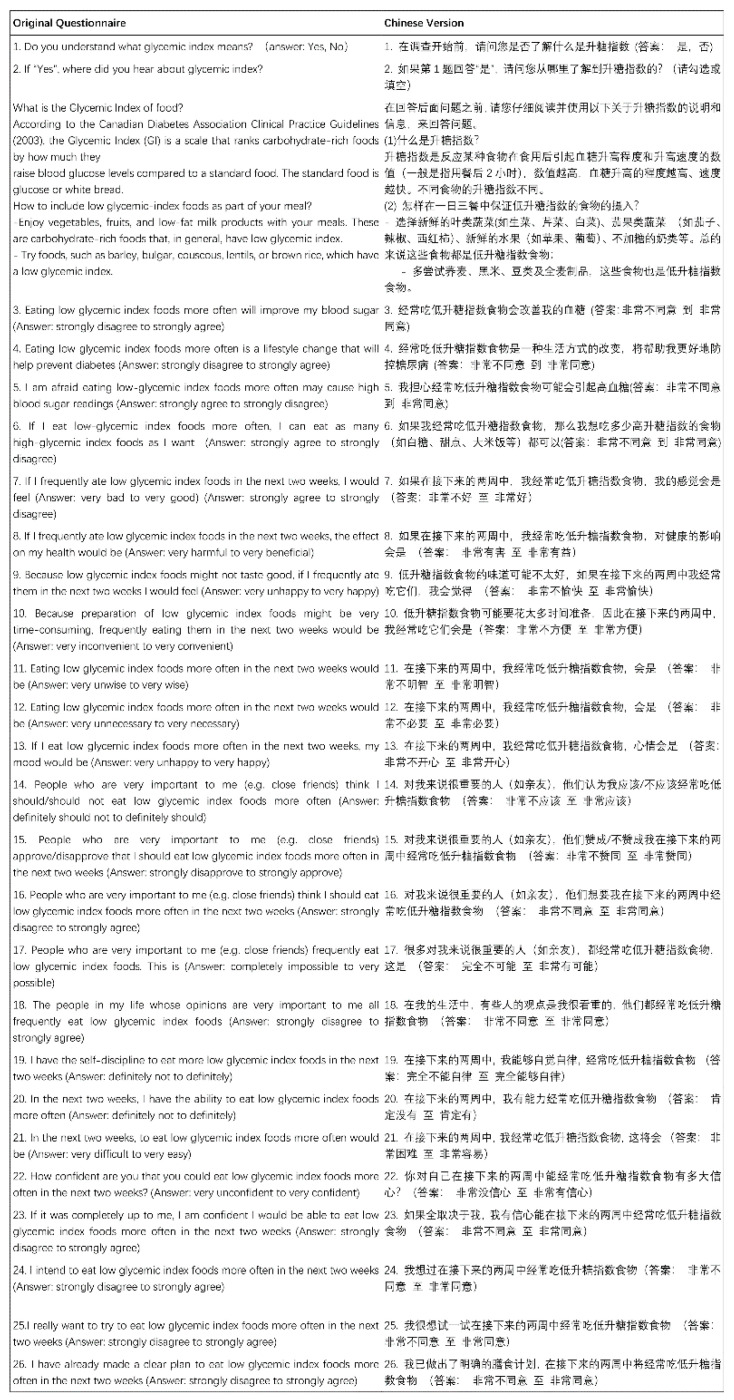
The original version and Chinese version of the questionnaire.

This study consisted of two phases: (1) cross-cultural adaptation and validation of the CIELQ; and (2) application of the CIELQ (Figure 1). In phase one, we translated and culturally adapted the IELQ using an instrument proposed by the WHO [23]. Then, 50 adult Chinese women with GDM (*n* = 25) or with a history of GDM (*n* = 25) were invited to complete the pre-testing task. Amendments were made according to the pre-testing results. The original English version and translated Chinese version are provided in Figure A1. Then, another 230 nondiabetic, nonpregnant women with a history of GDM were enrolled in the validation of CIELQ. One participant was excluded for lacking CIELQ data, thus 229 participants remained for the final analysis of phase one. In phase two, we excluded 48 women who had been diagnosed with T2DM from the baseline group (*N* = 465), and 417 participants remained for the analysis of phase two.

### 2.3. Data Collection

#### 2.3.1. Assessment of the Intention to Eat Low-GI Foods

The CIELQ was applied to assess the intention to eat low-GI foods. After the translation and cultural adaptation of the IELQ, we developed the CIELQ, which consisted of 26 items and evaluated seven factors: knowledge, instrumental attitude, descriptive attitude, subjective norms, descriptive norms, PBC and behavior intention. The processes for validating and applying the CIELQ were performed by trained researchers.

#### 2.3.2. Assessment of Glycemic Status

The glycemic status of the participants was measured using fasting plasma glucose (FPG) levels and the 2-h 75-g oral glucose tolerance test (2h-OGTT) in a local hospital. All participants fasted overnight for at least 8 h before the assessment of their glycemic status. The cutoff point for normal/abnormal glycemic status was 6.1 mmol/L for FPG and 7.8 mmol/L for the 2h-OGTT [24].

#### 2.3.3. Anthropometric Measurements

Anthropometric measurements were performed by trained nurses in local hospitals. Height and weight were measured with the participant barefooted and lightly dressed. Height was measured using a calibrated scale at head level with the participant standing barefoot and recorded to the nearest centimeter. Weight and body composition were measured using a body composition analyzer (BC-718. Tanita, Tokyo, Japan). Weight was recorded to the nearest 0.1 kg. Body composition covariates, such as percentage of body fat and basal metabolism were determined from the results of the body composition analyzer. BMI was calculated and categorized into four levels: underweight (<18.5 kg/m^2^), normal (18.5–23.9 kg/m^2^), overweight (24.0–27.9 kg/m^2^), or obese (≥28 kg/m^2^) [25]. Waist circumference was measured using a flexible tape at the level of the umbilicus at the end of expiration and categorized into normal (<80 cm), high (80–90 cm), or very high (≥90 cm) [25].

#### 2.3.4. Dietary Measurements

Dietary intake was measured using the 24-h recalls over three consecutive days method by trained researchers [26]. In face-to-face interviews, participants were asked to recall and to report their food and beverage intake in the preceding 24 h. This was done on three consecutive days, and one day included a weekend day. A standard questionnaire was applied to record the information.

#### 2.3.5. Physical Activity Measurements

Physical activity was self-reported by the participants using the International Physical Activity Questionnaire Short Form (IPAQ-SF) [27]. The IPAQ-SF comprises 4 generic items, collecting information on the time spent on vigorous activities, moderate activities, walking, and sitting over 7 days. Then, the participants’ information was used to calculate the metabolic equivalent task (MET) level and categorical score.

#### 2.3.6. Other Covariates

A self-developed, standard questionnaire was applied to collect demographical information, history of disease, and gestational information, including age, ethnicity, education level, family monthly income, gravidity, parity, and history of diabetes of family members. Trained researchers collected detailed information in a face-to-face interview with each participant.

### 2.4. Statistical Analysis

Continuous variables were presented as the mean (standard deviation) or median (percentile). Categorical variables were summarized using counts and percentages. Internal consistency was estimated using Cronbach’s alpha coefficient and Guttman’s split-half coefficient [28]. The intra-class correlation coefficient (ICC) was used to assess test-retest reliability. The Keiser-Meyer-Olkin (KMO) and Bartlett’s test of sphericity values were used to assess the adequacy of the sample size and appropriateness of the factor analysis. SPSS Amos (version 21.0, IBM, New York, NY, USA) was used for the confirmatory factor analysis (CFA) and structural equation modeling (SEM) using the maximum-likelihood estimation method [29].

Multivariable linear regression was applied to identify the potential factors that influenced the CIELQ score. Multivariable logistic regression was conducted to explore the association between the CIELQ score and glycemic status. Unless specified otherwise, data analyses were performed using IBM SPSS (version 22.0); a *p* value < 0.05 was considered to be statistically significant.

## 3. Results

### 3.1. Cross-Cultural Adaptation and Validation of the CIELQ and Its Application

A total of 229 participants were included in the validation process for the CIELQ. The characteristics of the participants are presented in Table A1. During the first run of the reliability test on 24 items (Q3 to Q26), Cronbach’s alpha was 0.928 for the whole questionnaire. However, for the domain of knowledge (Q3 to Q6), the value was only 0.523 (Cronbach’s alpha for Q3 and Q4 was 0.782). Because we assumed that the knowledge scale would improve by removing items 5 and 6, we retested the reliability for the remaining 22 items (Q5 and Q6 excluded). The Cronbach’s alpha value for the whole questionnaire was 0.945 in the second run, and the value ranged from 0.724 to 0.901 for each domain (Table A2). Guttman’s split-half reliability was 0.859.

**Figure A2:**
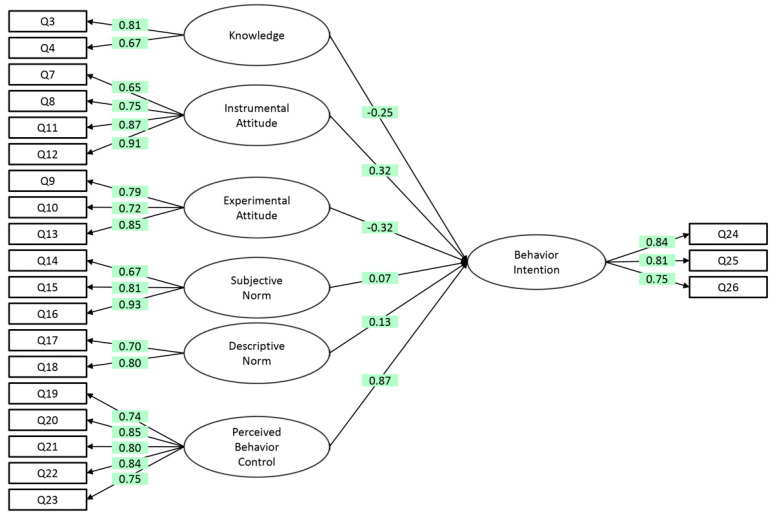
Structure of the CIELQ and standardized regression weights.

The KMO value was 0.918, and the *p* value of sphericity for Bartlett’s test was less than 0.005, indicating that the data were appropriate for factor analysis. The CFA results for the 7-factor model indicated acceptable fitness (Figure A2): the chi-square by degrees of freedom ratio (CMIN/DF) was 2.57, the goodness-of-fit index (GFI) was 0.84, the comparative fit index (CFI) was 0.91, the normed fit index (NFI) was 0.86, the standardized root mean-square residual (SRMR) was 0.076, and the root-mean-square error of approximation (RMSEA) was 0.083. When the correlation of Q7 and Q8 was added according to the modification index, the RMSEA decreased to 0.077. The average variance extracted (AVE) across the domains was 0.58 to 0.71. The structure of the questionnaire and standardized regression weights are shown in Figure A1. The original (IELQ) and translated version of the questionnaire (CIELQ) are provided in Figure A1.

### 3.2. Application of the CIELQ

#### 3.2.1. Characteristics of the Participants

A total of 417 participants were enrolled in the application phase of the CIELQ. The characteristics of the participants are shown in Table 1.

#### 3.2.2. CIELQ Score and Its Association with Covariates

The awareness of the GI was low among the study population (80 cases, 19.2%); the most frequent source of GI information was doctors (45 cases, 56.3%), followed by the internet (nine cases, 11.3%), books (five cases, 6.3%), other sources (five cases, 6.3%), and friends and family members (three cases, 3.8%).

We summed the scores for knowledge (Q3 and Q4), instrumental attitude (Q7, Q8, Q11, and Q12), experimental attitude (Q9, Q10, and Q13), subjective norms (Q14 to Q16), descriptive norms (Q17 and Q18), PBC (Q19 to Q23), behavior intention (Q24 to Q26), and the total CIELQ (Q3 to Q26, except for Q5 and Q6). The details of the scores of the specific factors are provided in Table 2.

The specific factors of the CIELQ were regressed onto demographic information, history of disease, gestational history, and other TPB factors using models 1–7 (Table A3). All models were significant (all *p* < 0.05), with *R*^2^ values ranging from 0.368 to 0.683. Education level was significantly associated with the scores for instrumental attitude, descriptive attitude, subjective norms, and behavior intention (all *p* < 0.05). Diabetes history of father or mother was a predictive variable for the scores for descriptive attitude, descriptive norms, and PBC (all *p* < 0.05). The TPB factors showed associations with each other. No evidence of multicollinearity (all variance inflation factors <2.692) or autocorrelation (range of the Durbin-Watson test: 1.977 to 2.278) was detected.

**Figure 2:**
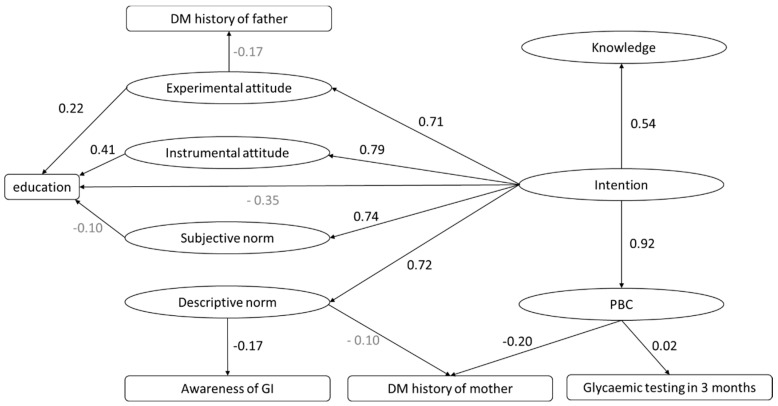
Simplified version of the structural equation modeling results. Path values: standardized coefficients of regression.

In model 7, the included variables accounted for 69.9% of the variability in behavior intention. All TPB variables were included in the SEM along with the significant covariates identified by previous linear regressions, including education, diabetes history of father, diabetes history of mother, having had a glycemic test in the last three months, and awareness of the GI (Figure 2). The fitness of the model was acceptable: CMIN/DF was 3.51, GFI = 0.82, CFI was 0.87, NFI was 0.82, SRMR was 0.067, and RMSEA was 0.081. All coefficients were statistically significant (*p* < 0.05) except for the following four pairs: education-subjective norm, DM history of mother-descriptive norms, DM history of father-experimental attitude, and education-intention.

All coefficients were statistically significant except for education-subjective norms, DM history of mother-descriptive norms, DM history of father-experimental attitude, and education-intention (statistically insignificant coefficients are in gray).

#### 3.2.3. CIELQ Score and Its Association with Glycemic Status

FPG level and glycemic level in the 2h-OGTT were regressed respectively onto demographic information, history of disease, gestational history, dietary intake factors, anthropometric information, physical activity, and TPB factors (Table 3).

No significant association was detected between FPG level and the scores for TPB factors. For the glycemic level in the 2h-OGTT, the score for instrumental attitude showed a positive association with the risk of a high level on the 2h-OGTT (OR = 1.330, *p* = 0.025), and the score for PBC showed a negative association with the risk for a high level on the 2h-OGTT (OR = 0.793, *p* = 0.035).

## 4. Discussion

To the best of our knowledge, this was the first study conducted among rural Chinese women to assess their intention to eat low-GI foods. We cross-culturally adapted and validated the questionnaire developed by Watanabe and colleagues [20], and the Chinese version was found to have a satisfactory fitness. In the primary application of the questionnaire, we found that the score for the TPB factors was associated with education level, diabetes history of family members, awareness of GI, and having had a glycemic test in the last three months. The scores for instrumental attitude and PBC were associated with the glycemic level in the 2h-OGTT.

The prevalence of GDM has increased in China in recent years. It was reported that the adjusted prevalence of gestational diabetes mellitus increased by 2.8 times from 1999 to 2008, from 2.4 to 6.8% [30]. A meta-analysis that summarized studies conducted after 2000 reported that the total incidence of GDM in mainland China was 14.8% (95% confidence interval 12.8–16.7%) [3]. Although the blood glucose levels of women with a history of GDM often normalize shortly after delivery, these women eventually have a substantially increased risk of developing T2DM later in life [31,32]. Fortunately, systematic postpartum lifestyle interventions can help to prevent the progression of GDM to T2DM [7]. Shyam and colleagues reported that low-GI diets successfully improved glucose tolerance in women with a previous history of GDM [8]. After a six month intervention, the women in the conventional healthy dietary recommendations group were found to have a higher blood glucose level on the 2h-OGTT, while the women in the low-GI diet group were found to have a lower blood glucose level on the 2h-OGTT (median (inter-quartile range): −0.2(2.8) vs. +0.8 (2.0) mmol/L, *p* = 0.025).

Over the past decades, the TPB has gained popularity and been widely applied for interpreting health-related behaviors, such as smoking, alcohol consumption, and weight reduction [15]. Watanabe et al. developed a questionnaire to assess intentions to eat low-GI foods (IELQ) in adults with diabetes based on the TPB and reported satisfactory fitness, validity, and reliability [20]. However, no such tool had been developed for rural Chinese women with a history of GDM. We conducted this study to adapt and validate the IELQ and test its fitness to the TPB in our population with the aim of providing evidence for designing an intervention program for rural Chinese women to prevent the progression of GDM to diabetes.

The culturally adapted CIELQ was found to have satisfactory performance in terms of reliability and validity. The Cronbach’s alpha value for the whole questionnaire was 0.945, and it ranged from 0.724 to 0.901 among the domains, similar to the results for the original questionnaire (0.78 to 0.93) [20]. Moreover, the ICC in the one week retest indicated good test-retest reliability. The CFA results indicated acceptable fitness of the tool; the CMIN/DF was 2.57, and the CFI was 0.91. By adding the correlation of Q7 and Q8 according to the modification index, the RMSEA value decreased from 0.083 to 0.077. This decrease in the RMSEA value might be explained by the logical correlation between Q7 and Q8. In this case, we hypothesized that people might feel good when eating low-GI foods because they realized that it was beneficial to their health.

After adapting the CIELQ to the study population, the results indicated that the CIELQ was a valid TPB-based instrument for assessing the intention to eat a low-GI diet among nondiabetic, nonpregnant Chinese women with a history of GDM. As shown in Figure 2, higher scores for knowledge, instrumental attitude, experimental attitude, subjective norms, and descriptive norms were associated with a higher score for the intention to eat low GI diets (all standardized coefficients of regression >0.6, except for knowledge [0.54]).

These findings are understandable because of the low awareness of the GI in this study population; less than one-fifth of the participants understood the meaning of the GI. Different from the study conducted by Watanabe and colleagues [20], which included patients with diabetes, we included nondiabetic, nonpregnant women with a history GDM in this study. The awareness of the GI in this study was much lower than the awareness reported for American adults with T2DM (46.7%) and for Canadian women with T2DM (63%) [33,34]. In addition, women who reported having knowledge of the GI concept also reported that the most frequent source of GI information was doctors (45 cases, 56.3%). This implied that diabetes education regarding the GI is insufficient among rural Chinese women with a history of GDM. The Obstetrics and Gynecology Branch of the Chinese Medical Association has advocated patient education and the postpartum follow-up of women with GDM [24], and further studies are needed to determine whether information on the GI should be emphasized during patient education and postpartum follow-up. In addition, education programs to develop awareness of the GI among rural women with a history of GDM are needed to increase the practice of eating a low-GI diet to prevent the potential long-term side effects of GDM.

Although a method using 24 h recalls over three consecutive days was applied to assess the dietary intake of the participants, we failed to calculate the GI for the mixed diet. Instead, we examined the correlation of the score for the Chinese Healthy Eating Index (CHEI) and that for the intention to eat low-GI foods. The CHEI is a valid tool for assessing diet quality in Chinese populations [35,36]. The CHEI score reflects dietary food intake by adding the scores for 17 types of food that are divided into two groups. Group one includes total grains, whole grains and mixed beans, tubers, total vegetables, dark vegetables, fruits, dairy, soybeans, fish and seafood, poultry, eggs, seeds, and nuts. Higher scores for group one indicate higher consumption levels of the foods in this group. Group two includes red meat, cooking oils, sodium, added sugars, and alcohol, which are encoded by a reverse method. We found that the CHEI score was marginally positively correlated with the score for intention in the CIELQ (Pearson correlation, r = 0.132, *p* = 0.008). Obviously, a high CHEI score indicates a healthy diet; this may serve as a rough estimation of the practice of eating low-GI foods. Admittedly, additional studies are needed to directly assess the association between the intention to eat low-GI foods and actually consuming low-GI foods.

We did not detect any significant association between FPG level and the score for TPB factors in the logistic regression. The *R*^2^ of the regression model was relatively small (0.177), indicating that many covariates that could influence FPG level were ignored, such as gene polymorphisms, diet, physical activity, dietary supplements, and sleep patterns [37,38,39,40,41,42].

Regarding the 2h-OGTT, women with a higher score for instrumental attitude were associated with a higher risk of impaired glucose tolerance (IGT, OR = 1.330, *p* = 0.025); women with a higher score for PBC were associated with a lower risk of IGT (OR = 0.793, *p* = 0.035). Again, the *R*^2^ of the regression model was relatively small (0.291). Previous studies have shown that behavior can be predicted by intention and PBC [15]. Moreover, instrumental attitude was positively associated with intention, and intention positively influences action [43]. The study conducted by Rhodes and colleagues showed that instrumental attitude contributed to perceived importance and concern regarding physical activities; it was significantly different among those with different physical activity engagement levels [44]. We initially hypothesized that the higher the scores for TPB variables were, the lower the risk of IGT. However, the association between instrumental attitude and risk of IGT in this study was counterintuitive. We suspected that this was due to bias resulting from a low awareness of the GI among the study population. Instrumental attitude refers to an individual’s evaluation of a behavior’s outcome, and is represented by adjective pairs such as valuable-worthless and harmful-beneficial [45]. To determine whether a behavior is beneficial, knowledge of the behavior and its relevant concepts are indispensable. However, more than 80% of the participants reported that they did not understand the GI. Thus, it was not surprising to find that the association between instrumental attitude and risk of IGT deviated from the anticipated result. PBC, which is defined as an individual’s perception of the ease or difficulty of performing a particular behavior, is an independent predictor of intention [46]. According to the TPB, intention along with PBC triggers the behavior [16]. Our findings were consistent with this structure; we hypothesized that women with a higher score for PBC were more willing to eat low-GI foods and benefit from glycemic control and be associated with a lower risk of IGT.

The CIELQ was found to have acceptable reliability and validity among nondiabetic, nonpregnant rural Chinese women with a history of GDM and a promising adaptability for other women of gestational age. However, our study still had several limitations. The awareness of the GI was low; in addition, we failed to assess participants’ knowledge of the GI in depth. As we mentioned above, the level of understanding of the GI could affect the attitude toward eating a low-GI diet and in turn influence other components in the TPB. Nondiabetic, nonpregnant rural Chinese women with a history of GDM were chosen for this study, but further studies in different populations are required before extrapolating the conclusions of this study to other groups. Nearly half of the participants included in this study were members of a minority ethnic group (43.9%); this is vastly different from the general trend for the Chinese population. According to the 2010 population census of China, the percentage of minority females aged 19–45 was 8.3% [47]. Further studies are needed to test the feasibility of the instrument in populations with a Han majority. Although HbA1c is a widely recognized indicator of the long-term glycemic status, we did not assess it in this study. In China, the HbA1c levels of patients are not widely used as diagnostic criteria because of the lack of standardization of HbA1c testing [24]. In addition, the inconsistencies in the cut-offs for the HbA1c level prevent distinguishing GDM patients from non-GDM patients. We did not directly collect information on the behavior of following a low-GI diet, such as the frequency that participants ate low-GI foods. In addition, information on some covariates that influence blood glucose levels was not considered. Further studies should include this information to better control for potential bias.

Comprehension of the predictors of the dietary behavior of women with a history of GMD is first needed before developing, implementing, and evaluating an effective nutritional intervention program and policy to prevent the progression of GDM to diabetes. Cultural background and characteristics of the subgroup population affect the reliability and validity of a questionnaire; cross-cultural adaptation and validation of the original questionnaire are essential. In this study, the fitness of TPB constructs to assess the intention to eat a low-GI diet provides a basic framework for health workers focused on women’s health in China to develop an intervention program. In particular, providing education on the concept of the GI needs to be emphasized when developing an intervention program. The association between the risk of IGT and PBC score indicates the importance of targeted learning objectives and program activities to ensure that the PBC of eating a low-GI diet is improved among rural Chinese women with a history of GDM.

## 5. Conclusions

The cross-cultural adaptation and validation of the CIELQ provided a valid instrument based on the TPB to assess the intention to eat a low-GI diet among rural Chinese women with a history of GDM. The awareness of the GI was poor among the study population. Women with a higher score for instrumental attitude were associated with a higher risk of IGT; women with a higher score for PBC were associated with a lower risk of IGT. Further studies based on the TPB to improve blood glycemic control should emphasize learning objectives and program activities related to providing education on the concept of the GI and improve the PBC of eating a low-GI diet.

## Figures and Tables

**Table 1:** Characteristic of the participants in the application of CIELQ (N = 417).

Variables		Cases	%
Age (years) ^a^			
	≤24	25	6%
	25–29	122	29.3%
	30–34	145	34.8%
	35–39	77	18.5%
	≥40	41	9.8%
Ethnicity ^b^			
	Han	233	55.9%
	Minority	183	43.9%
Education level			
	Primary school and below	4	1%
	Primary middle school	89	21.3%
	Senior middle school	120	28.8%
	College	114	27.3%
	Graduate and above	90	21.6%
Family monthly income (CNY) ^c^			
	≤999	9	2.2%
	1000–3000	98	23.5%
	≥3001	294	70.5%
BMI (kg/m^2^) ^d^			
	Underweight	17	4.1%
	Normal	209	50.1%
	Overweight	127	30.5%
	Obese	58	13.9%
Gravidity ^e^			
	1	108	25.9%
	2	96	23%
	3	96	23%
	4	67	16.1%
	≥5	49	11.8%
Parity ^f^			
	0	1	0.2%
	1	155	37.2%
	2	249	59.7%
	≥3	11	2.6%

Missing values: ^a^ seven cases in age, ^b^ one case in ethnicity, ^c^ 16 cases in family monthly income, ^d^ six cases in BMI, ^e^ one case in gravidity, ^f^ one case in gravidity.

**Table 2:** Summary of scores of specific factors of CIEQL.

Factors	N	Median (95% CI)
Knowledge (Q3,Q4)	412	12 (11.00–12.00)
Instrumental attitude (Q7, Q8, Q11 and Q12)	415	20 (20.00–21.00)
Experimental attitude (Q9, Q10 and Q13)	415	12 (12.00–13.00)
Subjective norm (Q14 to Q16)	414	16 (15.00–16.49)
Descriptive norm (Q17 and Q18)	415	9 (8.00–9.00)
Perceived behavioral control (Q19 to Q23)	413	24 (23.00–25.00)
Intention (Q24 to Q26)	414	15 (15.00–16.00)
Total score	398	106 (103.00–110.00)

CI, confidence interval.

**Table 3:** Multivariable logistic regression of glycemic status.

Dependent	Independent	Crude OR(95% CI)	*p*-Value	Adjusted OR(95% CI)	*p*-Value
FPG	Score of knowledge	0.988 (0.821–1.191)	0.903	-	-
	Score of instrumental attitude	1.023 (0.931–1.124)	0.634	-	-
	Score of experimental attitude	0.976 (0.870–1.096)	0.684	-	-
	Score of subjective norm	0.996 (0.891–1.113)	0.942	0.848 (0.640–1.043) ^a^	0.118
	Score of descriptive norm	0.801 (0.683–0.938)	0.006	-	-
	Score of PBC	0.960 (0.897–1.027)	0.236	-	-
	Score of behavior intention	0.968 (0.858–1.091)	0.591	-	-
	Score of total score	0.991 (0.970–1.012)	0.392	-	-
2h-OGTT	Score of knowledge	1.016 (0.893–1.156)	0.813	-	-
	Score of instrumental attitude	0.963 (0.901–1.029)	0.265	1.330 (1.036–1.708) ^b^	0.025
	Score of experimental attitude	1.047 (0.966–1.135)	0.260	0.837 (0.640–1.093) ^b^	0.191
	Score of subjective norm	0.965 (0.890–1.045)	0.381	-	-
	Score of descriptive norm	1.041 (0.931–1.164)	0.480	-	-
	Score of PBC	1.012 (0.964–1.062)	0.625	0.793 (0.640–0.983) ^b^	0.035
	Score of behavior intention	0.965 (0.883–1.054)	0.425	-	-
	Score of total score	1.000 (0.985–1.014)	0.974	-	-

FPG, fasting plasma glucose (<6.1 mmol/L = 1; 6.1–7.0 mmol/L = 2); 2h-OGTT, 2-h 75-g oral glucose tolerance test (<7.8 mmol/L = 1; 7.8–11.1 mmol/L = 2). ^a^ Covariates in the final equation: education level, basal metabolism, diabetes history of mother. ^b^ Covariates in the final equation: BMI category, family monthly income, waist circumference category, diabetes history of father, diabetes history of siblings, physical activity level, age group.

## Appendix A

**Table A1:** Characteristic of 229 participants in validation of CIEQL.

Variables			Cases		%
Age ^a^					
	≤24		11		4.8
	25–29		57		24.9
	30–34		81		35.4
	35–39		49		21.4
	≥40		21		9.2
Ethnicity					
	Han		91		39.7
	Minority		138		60.3
Education level					
	Primary school and below		3		1.3
	Primary middle school		38		16.6
	Senior middle school		55		24.0
	College		82		35.8
	Graduate and above		51		22.3
Family monthly income (CNY) ^b^					
	≤999		7		30.6
	1000–3000		64		27.9
	≥3001		145		63.3%
BMI (kg/m^2^) ^c^					
	Underweight		13		5.7
	Normal		111		48.5
	Overweight		70		30.6
	Obese		29		12.7
Gravidity ^d^					
	1		67		37.0
	2		50		27.6
	3		27		14.9
	4		19		10.5
	≥5		18		9.9
Parity ^e^					
	0	4		1.7	
	1	87		38.0	
	2	82		35.8	
	≥3	8		3.5	

Missing values: ^a^ 10 cases in age, ^b^ 13 cases in family monthly income, ^c^ 6 cases in BMI, ^d^ 48 cases in gravidity, ^e^ 48 cases in parity.

**Table A2:** Cronbach’s alpha value of CIELQ.

Dimension of Questionnaire	Cronbach’s Alpha
Total questionnaire	0.945
Knowledge	0.724
Instrumental attitude	0.871
Experiential attitude	0.823
Subjective norm	0.869
Descriptive norm	0.715
Perceived behaviour control	0.901
Behaviour intention	0.843

**Table A3:** Multivariable linear regression of specific factors of CIEQL.

Variables	Model 1		Model 2		Model 3		Model 4		Model 5		Model 6		Model 7	
	Beta	*p* Value	Beta	*p* Value	Beta	*p* Value	Beta	*p* Value	Beta	*p* Value	Beta	*p* Value	Beta	*p* Value
BMI category	0.007	0.875	0.018	0.617	0.039	0.322	0.089	0.022	0.009	0.841	−0.039	0.272	0.045	0.158
Age group	0.033	0.505	−0.007	0.866	0.081	0.054	−0.081	0.053	0.093	0.051	−0.013	0.724	0.052	0.130
Ethnicity	−0.01	0.835	−0.040	0.259	−0.009	0.815	−0.012	0.751	−0.012	0.779	0.042	0.231	−0.004	0.907
Education level	0.071	0.165	0.119	0.003 ^a^	−0.096	0.028 ^a^	−0.090	0.038 ^a^	0.039	0.432	−0.019	0.637	−0.089	0.013 ^a^
Family monthly income level	0.065	0.160	0.002	0.949	−0.037	0.348	0.013	0.747	0.064	0.154	−0.005	0.897	0.027	0.403
Diabetes history of father	0.035	0.442	−0.067	0.055	0.103	0.007 ^a^	−0.003	0.933	−0.011	0.805	−0.016	0.646	0.047	0.134
Diabetes history of mother	0.032	0.478	−0.020	0.579	0.056	0.144	−0.046	0.232	0.093	0.033 ^a^	−0.073	0.036 ^a^	−0.049	0.118
Diabetes history of siblings	−0.015	0.743	0.007	0.846	−0.044	0.250	0.006	0.883	−0.002	0.960	0.042	0.223	0.041	0.184
Gravidity	−0.062	0.270	0.053	0.223	0.022	0.646	−0.001	0.985	−0.004	0.947	−0.028	0.512	0.044	0.261
Parity	0.048	0.405	−0.032	0.483	−0.035	0.482	−0.033	0.504	0.062	0.267	0.031	0.491	−0.037	0.355
Awareness of GI	−0.027	0.561	0.035	0.336	−0.069	0.078	−0.023	0.556	−0.089	0.044 ^a^	0.04	0.257	−0.017	0.607
Glyceamic testing in 3 months	−0.07	0.132	0.018	0.614	−0.018	0.642	−0.081	0.037 ^a^	0.067	0.130	−0.019	0.598	0.016	0.619
Score of knowledge	-	-	0.232	0.000 ^a^	−0.093	0.046	0.109	0.017 ^a^	−0.063	0.224	0.139	0.001 ^a^	−0.017	0.650
Score of instrumental attitude	0.384	0.000 ^a^	-	-	0.347	0.000 ^a^	0.410	0.000 ^a^	0.084	0.210	0.079	0.139	0.129	0.008 ^a^
Score of experimental attitude	−0.127	0.046 ^a^	0.287	0.000 ^a^	-	-	−0.038	0.485	−0.003	0.957	0.412	0.000 ^a^	−0.133	0.003 ^a^
Score of subjective norm	0.152	0.017 ^a^	0.345	0.000 ^a^	−0.038	0.485	-	-	0.238	0.000 ^a^	0.153	0.002 ^a^	0.153	0.001 ^a^
Score of descriptive norm	−0.069	0.224	0.055	0.210	−0.003	0.957	0.185	0.000 ^a^	-	-	0.261	0.000 ^a^	0.116	0.003 ^a^
Score of PBC	0.236	0.001 ^a^	0.081	0.139	0.513	0.000 ^a^	0.187	0.002 ^a^	0.410	0.000 ^a^	-	-	0.636	0.000 ^a^

The dependent variables in Model 1 to Model 7 were score of knowledge, score of instrumental attitude, score of descriptive attitude, score of subjective norm, score of descriptive norm, score of PBC, score of behavior intention, respectively. ^a^ Statistically significant.
